# Reduction in hospital-wide mortality after implementation of a rapidresponse team: a long-term cohort study

**DOI:** 10.1186/cc10547

**Published:** 2011-11-15

**Authors:** Jeremy R Beitler, Nate Link, Douglas B Bails, Kelli Hurdle, David H Chong

**Affiliations:** 1 Pulmonary and Critical Care Unit, Massachusetts General Hospital, 55 Fruit Street, BUL-148, Boston, Massachusetts 02114, USA; 2 Department of Medicine, New York University School of Medicine, 550 First Avenue, NBV 16N1, New York, New York 10016, USA; 3 Faculty Group Practice, New York University Langone Medical Center, 1 Park Avenue, 10th Floor, New York, New York 10016, USA; 4 Division of Pulmonary, Allergy, and Critical Care Medicine, Columbia University College of Physicians and Surgeons, 622 West 168th Street, New York, New York 10032, USA

## Abstract

**Introduction:**

Rapid response teams (RRTs) have been shown to reduce cardiopulmonary arrests outside the intensive care unit (ICU). Yet the utility of RRTs remains in question, as most large studies have failed to demonstrate a significant reduction in hospital-wide mortality after RRT implementation.

**Methods:**

A cohort design with historical controls was used to determine the effect on hospital-wide mortality of an RRT in which clinical judgment, in addition to vital-signs criteria, was widely promoted as a key trigger for activation. All nonprisoner patients admitted to a tertiary referral public teaching hospital from 2003 through 2008 were included. In total, 77, 021 admissions before RRT implementation (2003 through 2005) and 79, 013 admissions after RRT implementation (2006 through 2008) were evaluated. The *a priori *primary outcome was unadjusted hospital-wide mortality. A Poisson regression model was then used to adjust for hospital-wide mortality trends over time. Secondary outcomes defined *a priori *were unadjusted out-of-ICU mortality and out-of-ICU cardiopulmonary-arrest codes.

**Results:**

In total, 855 inpatient RRTs (10.8 per 1, 000 hospital-wide discharges) were activated during the 3-year postintervention period. Forty-seven percent of RRTs were activated for reasons of clinical judgment. Hospital-wide mortality decreased from 15.50 to 13.74 deaths per 1, 000 discharges after RRT implementation (relative risk, 0.887; 95% confidence interval (CI), 0.817 to 0.963; *P *= 0.004). After adjusting for inpatient mortality trends over time, the reduction in hospital-wide mortality remained statistically significant (relative risk, 0.825; 95% CI, 0.694 to 0.981; *P *= 0.029). Out-of-ICU mortality decreased from 7.08 to 4.61 deaths per 1, 000 discharges (relative risk, 0.651; 95% CI, 0.570 to 0.743; *P *< 0.001). Out-of-ICU cardiopulmonary-arrest codes decreased from 3.28 to 1.62 codes per 1, 000 discharges (relative risk, 0.493; 95% CI, 0.399 to 0.610; *P *< 0.001).

**Conclusions:**

Implementation of an RRT in which clinical judgment, in addition to vital-signs criteria, was widely cited as a rationale for activation, was associated with a significant reduction in hospital-wide mortality, out-of-ICU mortality, and out-of-ICU cardiopulmonary-arrest codes. The frequent use of clinical judgment as a criterion for RRT activation was associated with high RRT utilization.

## Introduction

Adverse events are estimated to occur in 3% to 18% of hospitalizations in the United States, Canada, Europe, and Australia [1-9]. The degree to which these adverse events, including cardiopulmonary arrest and unexpected death, are preventable remains unclear. Clinical signs of deterioration, including vital-sign derangements and mental-status changes, are present at least 8 hours before most inpatient cardiopulmonary arrests [10-12]. Despite the ubiquity of cardiopulmonary-arrest teams, survival to discharge after in-hospital cardiopulmonary resuscitation remains only 7% to 26% [13-17]. This has led to attempts to identify early premonitory signs of arrest and to intervene promptly to prevent such adverse outcomes during hospitalization.

In response, the Institute for Healthcare Improvement in the United States [18], Department of Health in the United Kingdom [19], Commission on Safety and Quality in Health Care in Australia [20], and several other national patient-safety organizations have recommended adopting rapid response teams (RRTs) to reduce inpatient morbidity and mortality. The RRT, also known as a medical emergency team or critical care outreach team, is a multidisciplinary group of hospital personnel that responds promptly to acutely deteriorating inpatients outside the intensive care unit (ICU).

RRTs are typically activated once predefined vital-sign derangements are met. Such RRTs previously have been shown to reduce out-of-ICU cardiopulmonary arrests [21-25]. Given the low probability of survival to discharge after inpatient cardiopulmonary arrest, the potential impact of RRTs on hospital-wide mortality would seem great. Yet several previous studies have failed to demonstrate a significant reduction in adult hospital-wide mortality after RRT implementation [23,26-28], including three of the four largest studies evaluating hospital-wide mortality to date, with between 25, 000 and 68, 000 patients in the postintervention group [23,27,28]. Moreover, a recent meta-analysis of 17 studies, including more than 400, 000 patients, failed to find a reduction in overall hospital-wide mortality or adult mortality, although a significant reduction in pediatric mortality was observed [29]. Although one recent large study [30], including 73, 000 patients in the postintervention group, and few small short-term studies [21,22,24,31] have found a significant reduction in hospital-wide mortality after RRT implementation, little consensus exists on the true mortality benefit, if any, afforded by RRTs.

Failure of most RRTs to reduce hospital-wide mortality is often attributed to underutilization and delays in activation of the RRT once predefined vital-sign derangements are met [26,27,32-35]. We postulated that an RRT emphasizing clinical judgment, in addition to vital-signs criteria as a key trigger for activation, may increase RRT utilization and thus more effectively identify early the patients who are clinically deteriorating, leading to timelier interventions and improved outcomes.

To assess the impact of an RRT in which clinical judgment was widely promoted as an additional central criterion for activation, we conducted one of the largest and longest studies to date of hospital-wide mortality after RRT implementation. Additionally, we examined the impact of the RRT on out-of-ICU mortality and out-of-ICU cardiopulmonary-arrest codes.

## Materials and methods

### Study design

A cohort design with historical controls was used to determine the effect of the RRT on hospital-wide mortality at Bellevue Hospital Center, an 809-bed tertiary referral public teaching hospital in New York City. The distribution of hospital beds includes 246 medical beds, 72 surgical beds, 49 adult ICU beds, 37 pediatric beds, 20 pediatric ICU beds, 26 obstetrics beds, 46 rehabilitation beds, and 313 psychiatric beds.

Patients were included if admitted to any nonprisoner inpatient service between 2003 and 2008, regardless of their advanced-directive status. Prisoners were excluded to comply with Institutional Review Board requirements. In total, 77, 021 admissions before RRT implementation (January 1, 2003, through December 31, 2005) and 79, 013 admissions after RRT implementation (January 1, 2006, through December 31, 2008) were evaluated. Although RRTs were activated in both inpatient and outpatient settings during the postintervention period, only those RRTs activated for inpatients were considered.

Data on mortality, demographic characteristics, and case-mix index were collected prospectively in the hospital core patient database and retrieved for the study. (The case-mix index is a standardized measure of illness severity based on diagnosis, which is commonly used to determine insurance reimbursement for inpatient stays in U.S. hospitals.) Cardiopulmonary-arrest code and RRT data were collected prospectively for a quality-improvement initiative. After discharge, charts were reviewed to identify the diagnosis attributed by the primary medical team as prompting RRT activation and to identify advanced-directive status. The study was approved by the New York University School of Medicine and Bellevue Hospital-Health and Hospitals Corporation Institutional Review Boards, which waived the need for informed consent.

### Intervention

The RRT was led by the medical consult resident, a senior (postgraduate year 3) medical house officer who also led the cardiopulmonary-arrest code team, was certified in advanced cardiac life support, and typically had 4 to 6 months of prior critical care training. The team, which also included an ICU nurse, respiratory therapist, and patient transporter, was activated via the hospital page operator to report immediately to the bedside to direct management of the unstable patient. On nights and weekends, a nursing administrator joined the team for further support. Although not formal team members, additional medical house officers also frequently responded to RRT activations.

Clinical judgment that prompt assistance is needed at the bedside was emphasized repeatedly as an important criterion for RRT activation. Prespecified vital-signs criteria were also reviewed during training sessions, including pulse oximetry saturation less than 90%, respiratory rate less than eight or greater than 30 breaths per minute, systolic blood pressure less than 90 mm Hg, heart rate less than 40 or greater than 140 beats per minute, or change in heart rate greater than 30 beats per minute. However, staff were explicitly instructed to activate the RRT without hesitation for any appropriate degree of clinical concern without threat of repudiation or reprisal, regardless of whether the reviewed vital-signs criteria were met.

The RRT was first introduced in January 2006 to the medicine service through multidisciplinary sessions led jointly by senior physician and nurse members of the RRT development committee and attended by nurses, respiratory therapists, and medicine house staff on service. The RRT was expanded to all other adult inpatient, outpatient, testing, and administrative services between February and June 2006. Additional introductory sessions were held throughout the study period during new-staff orientations and at nursing leadership, patient safety, and quality improvement meetings. The RRT development committee reviewed monthly all out-of-ICU arrests and gave direct feedback to involved nurses and staff regarding use of the RRT. No additional staff were hired nor work hours extended to implement the new program.

### Main outcomes

The *a priori *primary outcome was hospital-wide mortality, defined as death before discharge. Secondary outcomes defined *a priori *were out-of-ICU mortality and out-of-ICU cardiopulmonary-arrest codes. A cardiopulmonary-arrest code was defined as active or impending cardiopulmonary arrest resulting in activation of the hospital-wide code team. Within-ICU cardiopulmonary-arrest codes were not included because ICU teams often handled cardiopulmonary arrests without activating the code system. These arrests were not captured in any hospital database.

### Statistical analysis

Demographic characteristics were analyzed by using the χ^2 ^test for categoric variables and the Student *t *test for continuous variables. To determine whether RRT implementation correlated with reductions in mortality and cardiopulmonary-arrest codes, the relative risk (RR) and 95% confidence intervals (CIs) were calculated by using Poisson regression. The difference in the absolute number of deaths was estimated by subtracting the observed number of postintervention deaths from the expected number of postintervention deaths (preintervention mortality rate multiplied by postintervention discharge volume). To adjust for year-to-year improvement in hospital-wide mortality that may have occurred independent of the intervention, a second Poisson model was constructed by including a linear term for time. For all analyses, the null hypothesis was evaluated by using a two-sided significance level of 0.05.

## Results

Small but statistically significant differences in demographic characteristics were found between the preintervention and postintervention groups (Table 1). Patients in the postintervention group on average had a higher acuity of illness, as measured by the case-mix index.

**Table 1:** Demographic characteristics of study population before and after rapid response team implementation

	**Pre-RRT**^**a**^ (*n *= 77, 021)	**Post-RRT**^**a**^ (*n *= 79, 013)	*P *value
Age, mean (SD)	40.9 (22.3)	42.0 (22.2)	< 0.001
Female	33, 595 (43.6)	33, 959 (43.0)	0.011
Race/Ethnicity			
Asian	10, 570 (13.7)	10, 431 (13.2)	
Black	18, 247 (23.7)	19, 359 (24.5)	
Hispanic	31, 146 (40.4)	31, 587 (40.0)	< 0.001
White	12, 995 (16.9)	13, 950 (17.7)	
Other	4, 063 (5.3)	3, 686 (4.7)	
Case-Mix Index	1.55	1.76	

RRT, rapid response team. ^a^Values are expressed as number (percentage), unless otherwise noted.

**Figure 1:**
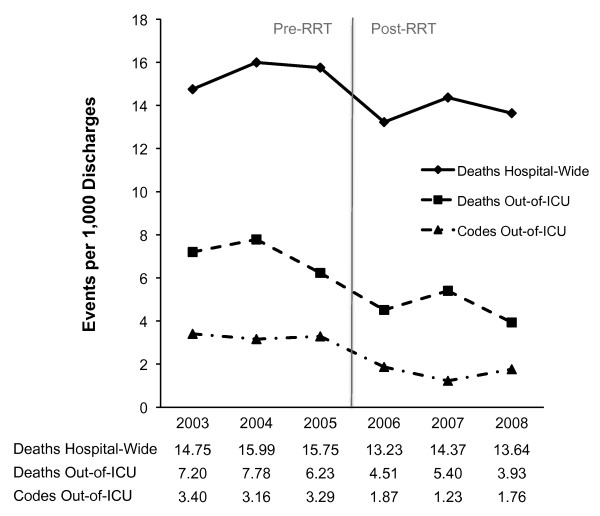
**Annual mortality and cardiopulmonary-arrest code rates before and after rapid response team (RRT) implementation**. The preintervention period was January 1, 2003, through December 31, 2005. The postintervention period was January 1, 2006, through December 31, 2008. The RRT was introduced over a 6-month period beginning January 2006. Patients admitted throughout introduction of the RRT were included in the postintervention group. Rates of hospital-wide deaths, out-of-ICU deaths, and out-of-ICU codes are provided as events per 1, 000 discharges.

Hospital-wide mortality significantly decreased from 15.50 to 13.74 deaths per 1, 000 discharges after RRT implementation (RR, 0.887; 95% CI, 0.817 to 0.963; *P *= 0.004) (Table 2, Figure 1). In absolute terms, the number of hospital deaths decreased by 139 after RRT implementation (from 1, 225 expected to 1, 086 observed deaths; 95% CI, 68 to 210). Adjusting for mortality trends over the 3-year period immediately before RRT implementation, hospital-wide mortality remained significantly lower after the intervention (RR, 0.825; 95% CI, 0.694 to 0.981; *P *= 0.029), translating to an adjusted estimate of 264 fewer hospital deaths (from 1, 350 expected to 1, 086 observed deaths; 95% CI, 1 to 598). Out-of-ICU mortality significantly decreased from 7.08 to 4.61 deaths per 1, 000 discharges (RR, 0.651; 95% CI, 0.570 to 0.743; *P *< 0.001).

**Table 2:** Mortality and cardiopulmonary arrest codes before and after rapid response team implementation

	Events **before RRT**^**a**^ (*n *= 77, 021)	Events **after RRT**^**a**^ (*n *= 79, 013)	**RRTs activated on hospital service**^**b**^ (*n *= 855)	Relative risk of event (95% CI)	*P *value
Deaths hospital-wide	1, 194 (15.50)	1, 086 (13.74)	--	0.887 (0.817-0.963)	0.004
Deaths by inpatient hospital service					
Medicine	462 (16.91)	303 (10.28)	637 (74.5)	0.608 (0.526-0.702)	< 0.001
Surgery	66 (5.65)	51 (4.32)	31 (3.6)	0.765 (0.531-1.102)	0.150
Pediatrics	13 (1.37)	8 (0.91)	2 (0.2)	0.664 (0.275-1.602)	0.362
Intensive care	649 (115.40)	722 (124.70)	12 (1.4)	1.081 (0.972-1.201)	0.152
Obstetrics	0 (0.00)	0 (0.00)	2 (0.2)	--	--
Rehabilitation	2 (1.07)	1 (0.55)	56 (6.5)	0.515 (0.047-5.681)	0.588
Psychiatry	2 (0.14)	1 (0.07)	71 (8.3)	0.476 (0.043-5.255)	0.545
Deaths out-of-ICU	545 (7.08)	364 (4.61)	--	0.651 (0.570-0.743)	< 0.001
Cardiopulmonary-arrest codes out-of-ICU	253 (3.28)	128 (1.62)	--	0.493 (0.399-0.610)	< 0.001

CI, confidence interval; RRT, rapid response team. ^a^Events refer exclusively to deaths or cardiopulmonary-arrest codes, as specified. Values are expressed as number (events per 1, 000 discharges). ^b^Values are expressed as number (percentage), which does not add to 100% because additional inpatient RRTs were activated in off-ward procedural suites.

Out-of-ICU cardiopulmonary-arrest codes significantly decreased from 3.28 to 1.62 codes per 1, 000 discharges after RRT implementation (RR, 0.493; 95% CI, 0.399 to 0.610; *P *< 0.001). Survival to discharge was able to be determined for 87% of patients who experienced an out-of-ICU cardiopulmonary-arrest code. Among these patients, a nonsignificant trend was noted toward increased post-code survival after RRT implementation (22.5% before implementation versus 41.4% after implementation; *P *= 0.13). As a result, out-of-ICU code-related mortality (defined as death before discharge after experiencing an out-of-ICU arrest code) significantly decreased from 2.55 to 0.95 deaths per 1, 000 discharges after RRT implementation (*P *< 0.001). In absolute terms, the number of out-of-ICU code-related deaths decreased by 126 (from 201 expected to 75 observed deaths; 95% CI, 99 to 154) after RRT implementation. Adjusting for time trends in mortality over the 3-year period before the intervention, out-of-ICU code-related deaths decreased by 217 (from 292 expected to 75 observed deaths; 95% CI, 99 to 431).

Considering outcomes across hospital services, mortality significantly decreased among patients admitted to the medicine service, from 16.91 to 10.28 deaths per 1, 000 medicine discharges after RRT implementation (RR, 0.608; 95% CI, 0.526 to 0.702; *P *< 0.001) (Table 2). Mortality in the ICU substantially increased from 115.40 to 124.70 deaths per 1, 000 ICU discharges, although this increase did not reach statistical significance (RR, 1.081; 95% CI, 0.972 to 1.201; *P *= 0.152). No other significant changes were found in service-specific mortality during the study period.

In total, 855 RRTs were activated for 740 inpatients during the postintervention period. Nearly three fourths of all RRTs were activated for inpatients on the medicine service (637 activations, 74.5%; Table 2). Nurses activated 728 RRTs (85.1%), followed by physicians (113 activations, 13.2%) and ancillary staff (14 activations, 1.6%) (Table 3). Forty-three percent of RRTs were activated for reasons other than prespecified vital-signs criteria. The leading reasons cited for RRT activation were "staff worried: clinical judgment that patient does not look right" (400 activations, 46.8%) and change in mental status (368 activations, 43.0%). Ninety-six RRTs (11.2%) were activated for patients with advanced directives specifying do-not-resuscitate (DNR) at the time of activation.

**Table 3:** Triggers for rapid response team activation

	No. (%) of RRT activations (*n *= 855)
**Position of activator calling RRT**	
Nurse	728 (85.1)
Physician	113 (13.2)
Physical or occupational therapist	7 (0.8)
Nonmedical staff	7 (0.8)
**Reasons cited for activating RRT**^**a**^	
RRT activated for reasons Including prespecified vital-signs criteria^b^	485 (56.7)
RRT activated for reasons other than prespecified vital-signs criteria^b^	370 (43.3)
Staff worried: clinical judgment that patient does not look right	400 (46.8)
Change in mental status	368 (43.0)
Pulse oximetry saturation < 90%	285 (33.3)
Systolic blood pressure < 90 mm Hg	165 (19.3)
Heart rate < 40, > 140, or change > 30 beats/minute	144 (16.8)
Respiratory rate < 8 or > 30 breaths/minute	117 (13.7)
Seizure-like activity	103 (12.0)
Unresponsive	65 (7.6)
Color change of patient	51 (6.0)
Hypoglycemia	30 (3.5)
Chest pain	29 (3.4)
Uncontrolled or excessive bleeding	25 (2.9)
Fall	19 (2.2)
Respiratory distress	17 (2.0)
Cold or pulseless extremity	9 (1.1)
Lightheaded or dizzy	8 (0.9)
Focal neurologic deficit	8 (0.9)
Unable to reach treating physician	4 (0.5)
Hypothermia	3 (0.4)
Other (one RRT only)	46 (5.4)
**Final diagnosis that immediately led to RRT activation**	
Seizure	120 (14.0)
Severe sepsis	102 (11.9)
Arrhythmia	83 (9.7)
Pneumonia	66 (7.7)
Aspiration	43 (5.0)
Syncope/Presyncope	41 (4.8)
Hemorrhage	39 (4.6)
Hypoglycemia	34 (4.0)
Asthma/COPD exacerbation	31 (3.6)
CHF exacerbation	30 (3.5)
Psychiatric	29 (3.4)
Malignancy	24 (2.8)
Opiate overdose	21 (2.5)
Medication-induced hypotension	20 (2.3)
Cirrhosis	15 (1.8)
Stroke	14 (1.6)
Atypical chest pain	12 (1.4)
Pulmonary embolus	12 (1.4)
Myocardial infarction	8 (0.9)
Hypertensive urgency/emergency	7 (0.8)
ETT, tracheostomy tube, or ventilator dysfunction	7 (0.8)
Medication-induced mental-status change	7 (0.8)
Dementia	6 (0.7)
Other (fewer than five RRTs)	70 (8.2)
Unknown	14 (1.6)

CHF, congestive heart failure; COPD, chronic obstructive pulmonary disease; ETT, endotracheal tube; RRT, rapid response team. ^a^Does not add to 100% because more than one reason could be cited as rationale for RRT activation. ^b^Prespecified vital-signs criteria: pulse oximetry saturation, < 90%; respiratory rate, < 8 or > 30 breaths/minute; systolic blood pressure, < 90 mm Hg; heart rate, < 40, > 140, or change, > 30 beats/minute.

The most common diagnoses that immediately led to RRT activation were seizure (120 activations, 14.0%), severe sepsis (102, 11.9%), arrhythmia (83, 9.7%), pneumonia (66, 7.7%), aspiration (43, 5.0%), and syncope/presyncope (41, 4.8%) (Table 3). The most common documented therapeutic interventions performed during RRTs were airway, respiratory, and cardiovascular support (Table 4). After RRT activation, 473 (55.3%) patients were transferred to a more highly monitored setting, including 371 (43.4%) to the ICU (Table 4). Three hundred fifty-two (41.2%) patients remained in the same room, two (0.2%) underwent emergency surgery, and one (0.1%), emergency cardiac catheterization. Twenty-four (2.8%) patients died during the RRT.

**Table 4:** Interventions performed by the rapid response team

	No. (%) of RRT activations (*n *= 855)
**Therapeutic interventions during RRT**	
Supplemental oxygen	538 (62.9)
Intravenous fluids	247 (28.9)
Intubation	140 (16.4)
Nebulizer	112 (13.1)
Suctioning	98 (11.5)
Antiseizure medication	83 (9.7)
Vasopressor	82 (9.6)
Antiarrhythmic	69 (8.1)
Furosemide	63 (7.4)
Glucose or dextrose	57 (6.7)
Opiates	35 (4.1)
Cardiopulmonary resuscitation	34 (4.0)
Cardioversion	29 (3.4)
Antibiotics	26 (3.0)
Naloxone	26 (3.0)
Blood products	24 (2.8)
Nitroglycerin	22 (2.6)
Steroids	21 (2.5)
Antihypertensive	18 (2.1)
Adjust ETT, tracheostomy, or ventilator	13 (1.5)
Other (fewer than 10 RRTs)	57 (6.7)
**Disposition immediately after RRT**	
	
Transfer to new ward or room type	473 (55.3)
Transfer to ICU	371 (43.4)
Transfer to observation room^a^	41 (4.8)
Transfer to telemetry	26 (3.0)
Transfer to medicine floor	19 (2.2)
Transfer to emergency department	11 (1.3)
Transfer to neurology ward	2 (0.2)
Transfer to pediatrics ward	2 (0.2)
Transfer to surgery ward	1 (0.1)
Remain in same ward or room type	352 (41.2)
Remain in ICU	12 (1.4)
Remain in observation room	17 (2.0)
Remain on telemetry ward	82 (9.6)
Remain on general service ward	241 (28.2)
Other disposition immediately after RRT	30 (3.5)
Emergency surgery	2 (0.2)
Emergency cardiac catheterization	1 (0.1)
Death during RRT	24 (2.8)
Unknown	3 (0.4)

ETT, endotracheal tube; RRT, rapid response team. ^a^Observation rooms are four-bed medical ward rooms with a nurse present inside the room at all times.

Among patients for whom an RRT was activated, 540 (73.0%) survived to discharge, including 36 (4.9%) with an advanced directive specifying DNR at the time of at least one RRT activation. One hundred seventy-seven (23.9%) patients receiving an RRT died before discharge, including 60 (8.1%) patients for whom advanced directives were for full resuscitation at death, and 117 (15.8%) who were DNR at death. Seventy patients who were DNR at death had advanced directives specifying full resuscitation at the time of RRT activation. For 23 (3.1%) patients, their status at discharge was unknown.

## Discussion

Implementation of the RRT was associated with a significant reduction in hospital-wide mortality. In the 3-year postintervention period, 139 fewer deaths occurred than would be expected from the preintervention mortality rate. Additionally, 132 fewer out-of-ICU codes and 126 fewer out-of-ICU code-related deaths were found after RRT implementation than would be expected from preintervention rates.

Attributing the reduction in hospital-wide mortality to the RRT is strongly supported by the concurrent 51% decrease in out-of-ICU cardiopulmonary arrest codes and 35% decrease in out-of-ICU mortality (Table 2). Despite a substantial increase in the case-mix index, which indicated a secular trend toward increasing severity of illness among admitted patients, hospital-wide mortality decreased by 1.76 deaths per 1, 000 discharges. This mortality reduction remained statistically significant after adjusting for trends over time, and it was driven by a 39% decrease in mortality on the medicine service, where 75% of the RRTs took place. A commensurate reduction of 1.66 out-of-ICU cardiopulmonary-arrest codes and 1.60 out-of-ICU code-related deaths per 1, 000 discharges occurred, supporting attribution of the hospital-wide mortality reduction to the prevention of out-of-ICU arrests. The decreases in hospital-wide mortality, out-of-ICU mortality, and out-of-ICU cardiopulmonary-arrest codes all coincided temporally with introduction of the RRT in 2006 (Figure 1).

Moreover, the reduction in hospital-wide mortality was nearly fully matched by a commensurate decrease in out-of-ICU codes and code-related mortality. The slightly greater reduction in hospital-wide mortality may be accounted for by additional lives saved during RRTs activated for patients with advanced directives specifying DNR. Any improvement in mortality among DNR patients due to the RRT would not be reflected by changes in code-related deaths, as DNR patients were explicitly excluded from the cardiopulmonary-arrest code response. Alternatively, this difference may be due to confounding influences that the study was not designed to capture.

The descriptive findings of RRT activations further support attribution of the reduction in hospital-wide mortality to the RRT. The most common diagnoses that immediately led to RRT activation--seizure, severe sepsis, arrhythmia, pneumonia, and aspiration--are high-acuity conditions that often require ICU-level care. The most common therapeutic interventions performed by the RRT centered on airway, respiratory, and cardiovascular support (Table 4). Furthermore, no other concomitant patient-safety or quality-improvement interventions were introduced during the study period to account for the improved clinical outcomes. Although introducing hospitalists and intensivists previously was shown to reduce inpatient mortality [36-39], no substantial changes in hospital staffing or work hours occurred during the study period.

Previously, most large studies assessing hospital-wide mortality failed to demonstrate reduced mortality after RRT implementation [23,27,28], as did a recent meta-analysis evaluating hospital-wide mortality of more than 400, 000 patients [29]. Two likely explanations exist for the mortality reduction seen here, but not in most previous RRT studies.

First, this RRT was widely used, with 10.8 activations per 1, 000 hospital-wide discharges and 21.6 activations per 1, 000 medicine service discharges. A comparable hospital-wide activation rate of 9.3 RRT activations per 1, 000 admissions was observed in the only other large study to date to find a significant reduction in postintervention hospital-wide mortality [30]. By contrast, two of the three previous largest negative studies reported lower RRT utilization of 2.5 [28] and 8.7 [27] activations per 1, 000 admissions. In the MERIT trial [27], the only large cluster-randomized study to date, just 41% of patients who had RRT-activation criteria present more than 15 minutes before an adverse event actually had an RRT activated. Yet, a subsequent *post hoc *analysis of MERIT [40] demonstrated a significant dose-response relation between rate of RRT activation and incidence of cardiac arrests and deaths.

Underutilization is commonly reported in other RRT studies and may minimize improvements in clinical outcomes gained with adopting an otherwise effective RRT [26,32,33,40]. Delayed activation similarly has been associated with increased mortality [34,35,40]. Whereas failure to activate an RRT promptly may reflect insufficient staff awareness, most studies highlight the great lengths taken to promote their system. Thus, failure of prompt activation may instead reflect reluctance by nurses, junior physicians, and allied health professionals to go outside the traditional hierarchic model for referrals of clinical management (that is, junior nurse to senior nurse to junior physician to senior physician), even for acutely decompensating patients who meet criteria for RRT activation [21,41].

In this case, several features of RRT design and hospital culture promoted greater utilization. First, the RRT was run by the medical consult resident, who was already widely recognized by nurses as leader of the cardiopulmonary-arrest code team. Conversely, introduction of a new and unfamiliar staff member, such as a senior attending or intensivist, to lead the team might have introduced a psychological barrier to nurses activating the RRT in borderline cases. Second, as a public teaching institution staffed by house-officer trainees and salaried teaching attending physicians, the hospital had a preexisting culture of shared responsibility for patient care, leading to wide acceptance of the RRT model by the physician and nurse staffs. Third, the training of hospital staff explicitly encouraged heavy use of the RRT with a low threshold for activation and emphasized clinical judgment of nurses as a key activation criterion, creating an avenue for nursing empowerment. As a result, nurses took ownership of the RRT, accounting for 85% of all RRT activations and a higher rate of hospital-wide utilization than found in most other studies.

A second plausible explanation for the reduction in hospital-wide mortality is the emphasis that was placed on clinical judgment as a key criterion for RRT activation during staff training sessions, which may have prompted earlier RRT activations before intractable clinical deterioration. Although most previous studies have included clinical judgment as an activation criterion, the RRT in these studies was only infrequently activated for this reason [22-24,26,27,31]. By contrast, 43% of RRTs in the present study were activated for reasons other than vital-sign derangements. Furthermore, vital signs--and in particular respiratory rate--are often inaccurately measured and recorded [42], which may lead to further underutilization of the RRT. Potential failures to trigger RRT activation through vital-signs criteria may have been circumvented by emphasizing clinical judgment, as reflected in the high rate of RRT activation for "staff worried: clinical judgment that patient does not look right."

Interpretation of these findings shares limitations similar to those of most other RRT studies. Common to any cohort study with historical controls, it is possible that improvements in mortality and code rates were due to differences in the preintervention and postintervention populations rather than to the RRT itself. However, the trend toward increased severity of illness throughout the study period, as measured by the case-mix index, likely would have increased the risk of death during the postintervention period. Also considered was whether improvements in mortality reflected a secular downward trend that predated the RRT, yet the mortality rate actually trended upward during the 3-year preintervention period, coinciding with the increasing case-mix index. Adjusting for this time trend thus increased the estimated postintervention mortality improvement. However, the adjusted results had much wider confidence intervals and therefore provide a less reliable point estimate, compared with the unadjusted results, of the decreased mortality after RRT implementation. Regardless, hospital-wide mortality remained significantly lower after RRT implementation after controlling for underlying trends in mortality over time.

Several outcomes measures, including out-of-ICU mortality and cardiopulmonary-arrest codes, could be favorably biased by excluding mortality of patients transferred to the ICU. This study instead focused on hospital-wide mortality, which avoids this bias by counting all deaths regardless of where they occur in the hospital. Still, other unmeasured confounders, which our study was not designed to capture, may have favorably biased the study results. For example, it is possible that the transfer of patients to outside hospice or other long-term care facilities increased during the study period. Yet, no changes to palliative care services occurred throughout the study period, and the improvement in overall mortality was nearly completely accounted for by the decrease in out-of-ICU codes and associated deaths.

The low rate of RRT activation and low baseline mortality among nonmedical services may limit generalizability of these results to hospitals with a large nonmedical patient composition or differences in service-specific mortality. Additionally, these findings reflect a single tertiary referral public teaching hospital's experience and may not be generalizable to nonteaching or lower-acuity hospitals. Finally, difficulty in standardizing clinical judgment as a criterion for RRT activation may limit generalizability across institutions with different levels of staff experience and out-of-ICU monitoring. It may be precisely this flexibility, to activate an RRT for any reasonable clinical judgment without threat of repudiation or reprisal, that resulted in high RRT utilization and led to the mortality reduction observed.

## Conclusions

To our knowledge, this is the largest study to date to demonstrate as its primary outcome a reduction in hospital-wide mortality associated with RRT implementation. An estimated 139 fewer deaths occurred hospital-wide over the 3-year period immediately after adoption of the RRT. In contrast to most other large studies, which have not demonstrated reduced mortality, this RRT was commonly activated for reasons of clinical judgment, in addition to vital-signs criteria, and featured a high rate of RRT activation. Additional studies are needed to confirm the effect of similarly designed and implemented RRTs in hospitals with different staffing models, staff cultures, and patient populations, and to identify the optimal team composition, activation criteria, and implementation strategy for RRTs.

## Key messages

• Implementation of a rapid response team (RRT) in which clinical judgment, in addition to vital-signs criteria, was widely cited as a rationale for activation was associated with an 11% decrease in hospital-wide mortality.

• In contrast to those in other published reports, 46.8% of RRTs in this study were activated for reasons of clinical judgment. Most previous studies have included clinical judgment as a criterion for activation, but the RRT in these studies was only infrequently activated for this reason.

• The regular use of clinical judgment as a criterion for RRT activation was associated with higher rates of RRT activation than found in other published reports of RRTs.

## Abbreviations

CHF: congestive heart failure; CI: confidence interval; COPD: chronic obstructive pulmonary disease; DNR: do-not-resuscitate; ETT: endotracheal tube; ICU: intensive care unit; RRT: rapid response team.

## Competing interests

The authors declare that they have no competing interests.

## Authors' contributions

JRB, NL, DBB, KH, and DHC designed the study. JRB collected the data. JRB and NL completed the data analysis. JRB, NL, DBB, KH, and DHC interpreted the results. JRB drafted the manuscript. JRB, NL, DBB, KH, and DHC critically reviewed the manuscript. JRB and NL completed revisions. All authors read and approved the final manuscript.

## Additional information

The findings of this study were presented in part at the American Thoracic Society International Conference, May 18, 2010, in New Orleans, Louisiana, USA.
